# Unexpected effects of different genetic backgrounds on identification of genomic rearrangements via whole-genome next generation sequencing

**DOI:** 10.1186/s12864-016-3153-9

**Published:** 2016-10-21

**Authors:** Zhangguo Chen, Katherine Gowan, Sonia M. Leach, Sawanee S. Viboolsittiseri, Ameet K. Mishra, Tanya Kadoishi, Katrina Diener, Bifeng Gao, Kenneth Jones, Jing H. Wang

**Affiliations:** 1 Department of Immunology and Microbiology, University of Colorado, Anschutz Medical Campus, 12800 E, 19th Ave, Mail Stop 8333, Aurora, CO 80045 USA; 2 Department of Biomedical Research, National Jewish Health, Denver, CO 80206 USA; 3 Department of Biochemistry and Molecular Genetics, University of Colorado, Anschutz Medical Campus, Aurora, CO 80045 USA; 4 Integrated Center for Genes, Environment and Health, National Jewish Health, Denver, CO 80206 USA; 5 Genomic and Microarray Core, University of Colorado, Anschutz Medical Campus, Aurora, CO 80045 USA

**Keywords:** Whole genome next generation sequencing, Genomic instability, B cell lymphoma, Different genetic background, de novo assembly

## Abstract

**Background:**

Whole genome next generation sequencing (NGS) is increasingly employed to detect genomic rearrangements in cancer genomes, especially in lymphoid malignancies. We recently established a unique mouse model by specifically deleting a key non-homologous end-joining DNA repair gene, *Xrcc4*, and a cell cycle checkpoint gene, *Trp53*, in germinal center B cells. This mouse model spontaneously develops mature B cell lymphomas (termed G1XP lymphomas).

**Results:**

Here, we attempt to employ whole genome NGS to identify novel structural rearrangements, in particular inter-chromosomal translocations (CTXs), in these G1XP lymphomas. We sequenced six lymphoma samples, aligned our NGS data with mouse reference genome (in C57BL/6J (B6) background) and identified CTXs using CREST algorithm. Surprisingly, we detected widespread CTXs in both lymphomas and wildtype control samples, majority of which were false positive and attributable to different genetic backgrounds. In addition, we validated our NGS pipeline by sequencing multiple control samples from distinct tissues of different genetic backgrounds of mouse (B6 vs non-B6). Lastly, our studies showed that widespread false positive CTXs can be generated by simply aligning sequences from different genetic backgrounds of mouse.

**Conclusions:**

We conclude that mapping and alignment with reference genome might not be a preferred method for analyzing whole-genome NGS data obtained from a genetic background different from reference genome. Given the complex genetic background of different mouse strains or the heterogeneity of cancer genomes in human patients, in order to minimize such systematic artifacts and uncover novel CTXs, a preferred method might be *de novo* assembly of personalized normal control genome and cancer cell genome, instead of mapping and aligning NGS data to mouse or human reference genome. Thus, our studies have critical impact on the manner of data analysis for cancer genomics.

**Electronic supplementary material:**

The online version of this article (doi:10.1186/s12864-016-3153-9) contains supplementary material, which is available to authorized users.

## Background

Multiple mechanisms operate in B lymphocytes that intrinsically generate DNA double-stranded breaks (DSBs) or mutations during B cell differentiation [1, 2]. Mature B lymphocytes undergo class switch recombination (CSR) and somatic hypermutation (SHM), in specialized secondary lymphoid structures termed germinal centers (GCs) [3], during which DSBs or mutations are introduced to immunoglobulin (Ig) loci [4]. Apart from programmed DSBs, B lymphocytes harbor general DSBs caused by genotoxic agents [5]. Non-homologous end-joining (NHEJ) is the predominant DSB repair pathway in mammalian cells, operating in all phases of cell cycle [5]. We and others have clearly shown that NHEJ plays an essential role in maintaining genome stability [6–13].

XRCC4, Lig4, and possibly XLF, form a complex to catalyze the end-ligation step of NHEJ [5, 14]. Conditional deletion of *Xrcc4* or *Lig4* in peripheral B cells reduces the CSR level and causes a high level of chromosomal breaks and translocations at the *Igh* locus [12, 13]. In addition, we previously showed that conditionally deleting *Xrcc4* in *p53*-deficient peripheral B cells led to the development of surface Ig negative lymphomas from editing and switching B cells [11]. We identified clonal translocations involving *Igh* and *IgL* loci in these lymphomas using conventional cytogenetic techniques such as fluorescence in situ hybridization (FISH) [11]. However, it has not been investigated whether NHEJ defects impose a global impact on the overall stability of mature B cell genomes.

Chromosomal translocations have been long recognized to be cancer-driven in hematological malignancies [15]. For example, the classic *Igh-c-myc* translocation is the hallmark of Burkitt’s lymphomas and BCR-ABL translocation underlies chronic myelogenous leukemia [16, 17]. Many of such chromosomal translocations are reciprocal balanced translocations, which do not result in a change in DNA dosage, or involve two or more chromosomal cross-overs, making detection of such translocations technically challenging. Due to the experimental difficulties of detecting such translocations, this class of structural variation remains largely unstudied [18].

Whole genome next generation sequencing (NGS) approach potentially provides an exciting opportunity to discover chromosomal rearrangements in cancer genomes [19]. Thus, we attempt to decipher the mechanisms promoting the genomic complexity in NHEJ deficient B cell lymphomas using whole genome NGS. Surprisingly, we found that widespread false positive genomic rearrangements can be generated by simply aligning NGS data from mouse ﻿s﻿trains whose genetic background is different from mouse reference genome (B6). Given that many of cancer genome sequencing studies routinely performed mapping and alignment of NGS data to human reference genome [19] and that human populations have different genetic background, we suggest that alignment of NGS data from individual cancer genomes to the published human reference genome may overestimate cancer genome instability. Thus, our results have critical impact on the manner of data analysis for characterizing genomic complexity in cancer genomes, which we discuss in great detail.

## Methods

### Generation of mouse models

Cγ1Cre knock-in (KI) mice [20], *Xrcc4* [13] or *p53* [21] conditional knock-out (KO) mice were generated previously. These mice were in mixed genetic background of C57BL/6J, 129/Ola and FVB/N [20, 21]. Once the desired genotypes were obtained by intercrossing the three different strains, G1XP mice were inbred among them for at least seven generations to establish the cohort for tumor study. Wt C57BL/6J (B6) mice were purchased from Jackson Laboratory. Animal work was approved by the Institutional Animal Care and Use Committee of University of Colorado Anschutz Medical Campus (Aurora, CO) and National Jewish Health (Denver, CO).

### NGS library preparation, sequencing platform and data analysis

Tumor DNA samples were employed to generate the NGS paired-end library using the standard TruSeq DNA library preparation kit (Illumina, San Diego, CA). The libraries were subjected to whole genome sequencing on the Illumina Hi-Seq 2000 platform (pair-ended, 2 × 100 bp per read), with coverages ranging between 30× and 40× for the six sequenced tumor samples, labelled 46J, 90J, 119J, 125J, 196J, and 202J for tumor T1-T6, respectively. DNA samples were isolated from wt primary B cells (see below) or kidney in the same genetic background as the tumor samples or in pure B6 background. The libraries were subjected to whole genome sequencing on the Illumina Hi-Seq 2000 platform (pair-ended, 2 × 150 bp per read). The mean Phred quality scores of ten sequenced samples are: 46J (36.20), 90J (36.70), 119J (36.92), 125J (36.31), 196J (36.55), 202J (37.07), Wt Control 1 (35.16), Wt Control 2 (35.22), Wt kidney (34.33), and Wt B6 (35.38). All samples are in the same genetic background (non-B6) except Wt B6 which is in pure C57BL/6J background from Jackson Laboratory.

The whole genome NGS raw data was first aligned to mouse mm9 reference sequences (B6 background), then, we employed CREST (‘clipping reveals structure’) to detect structural variation (SV) including deletions (DEL), inter-chromosomal translocations (CTXs) and others. CREST is an algorithm that uses NGS reads with partial alignments to a reference genome to directly map SV at the nucleotide level of resolution [22]. We have tested several algorithms for SV detection, and CREST appears to be the one that performs robustly and has a relatively high predictive accuracy. In addition, CREST has been employed to analyze NGS data of human leukemia or lymphoma samples [22], or NGS data obtained from other types of human cancers using the same Illumina HiSeq 2000 platform as we did, which indeed detected SVs [23, 24]. Therefore, we used this algorithm to identify SVs in our mouse lymphoma samples. After SV detection by CREST, candidate SV calls were extensively filtered for those most likely to be “true” novel rearrangements. Each variant was required to be unique to a single sample sequenced including six tumor samples and four control samples, and to have evidence of soft-clipping reads at each contributing breakpoint end. Any rearrangements involving the mitochondrion, the Y chromosome or unmapped contigs were excluded from further analysis. Thus, all CTXs identified and depicted in the Circo plots are distinct, non-overlapping events that are unique across all tissues including tumors and normal controls. The NGS data of the 129S1/SvImJ genome was downloaded from Sanger Institute’s website (http://www.sanger.ac.uk/science/data/mouse-genomes-project) [25], aligned to mouse reference genome (mm9) and analyzed by CREST for SV detection. Circos plots were generated using the software described previously [26]. Lastly, we were able to confirm the occurrence of some of NGS-identified CTXs in tumors with independent methodology (e.g. FISH or PCR).

### Primary B cell culture and immunization

Splenic B cells were isolated from wt naïve mice in either pure B6 or non-B6 genetic background that is the same as G1XP lymphomas. B cells were purified by negative selection kit (Stem Cell Technologies, Canada), activated with anti-CD40 and IL-4 as described previously [27], and collected 4 days after culture for genomic DNA isolation which were subject to NGS. *In vivo* immunization and GC B cell isolation were performed as described previously [27]. The GC B cells were isolated from non-B6 genetic background that is the same as G1XP lymphomas.

## Results

### Detection of widespread inter-chromosomal translocations (CTXs) in G1XP lymphomas

**Fig. 1:**
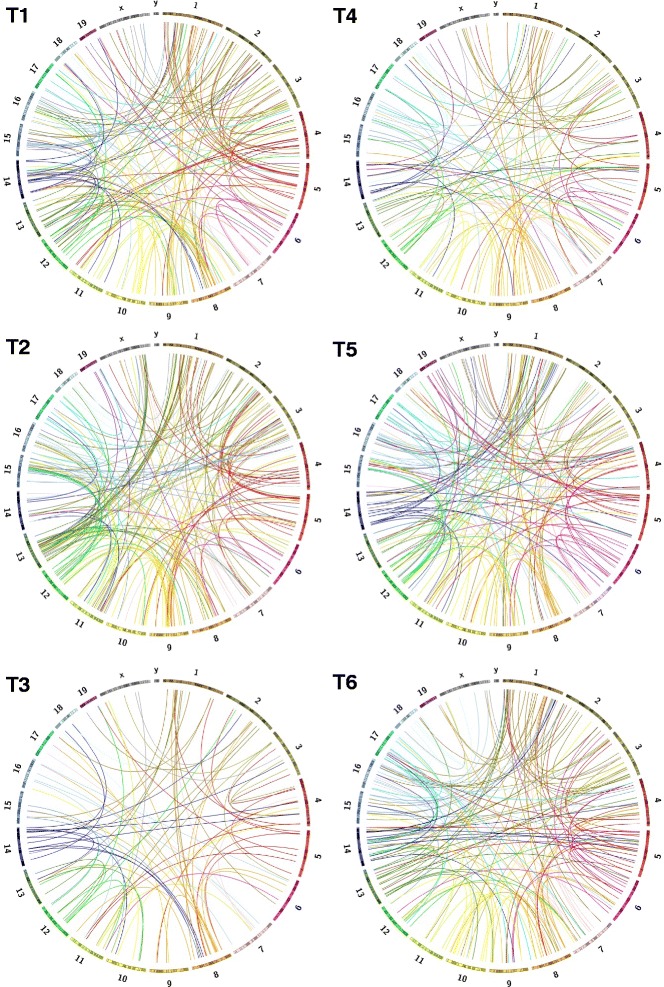
Dramatically increased genomic complexity in G1XP lymphomas. All of the unique inter-chromosomal translocation (CTX) events are shown as Circos plots for six sequenced G1XP lymphoma samples. Each color-coded bar represents an individual chromosome with its specific banding patterns shown. Each color-coded line represents a CTX event originating from that particular chromosome

We recently established a novel experimental model distinct from previous ones by deleting *Xrcc4* and *Trp53* in a subset of activated B lymphocytes via Cγ1cre, which predisposes B cells to lymphomagenesis [28]. We termed these *Xrcc4/Trp53* deficient B lineage tumors G1XP lymphomas, and employed the whole genome NGS technique to globally assess the level of genomic complexity in six G1XP lymphoma samples. The NGS data was mapped to mouse reference genome (mm9) and analyzed via CREST algorithm for structural variation (SV) detection [22] (see details in Methods). Our data revealed that G1XP lymphomas harbor an extremely high level of genomic complexity; in particular, deletions (thousands of events) and inter-chromosomal translocations (CTX) (hundreds of events) display the highest frequency observed in all six samples sequenced (data not shown). Circos plots showed that there were hundreds of unique CTXs involving almost all of the chromosomes in all six tumors (Fig. 1). In addition, the numbers of CTXs in each sample and chromosome coordinates of all CTXs were shown in Additional file 1: Table S1.

### Whole genome NGS of multiple control samples from the same genetic background as G1XP lymphomas

**Fig. 2:**
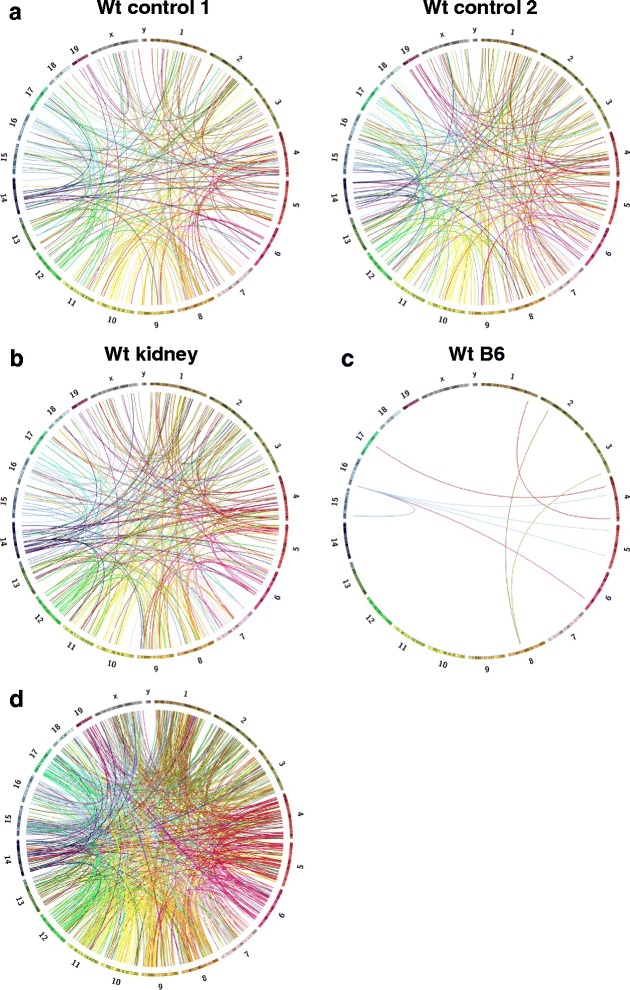
Dramatically increased genomic complexity caused by mixed genetic background. All of the unique CTX events are shown as Circos plots for two sequenced wt activated B cell samples (control 1 and 2, mixed genetic background) (**a**), wt kidney sample (control 1, mixed genetic background) (**b**), and wt activated B cell sample from pure B6 background (**c**). **d** Unique CTX events are shown as Circos plots from the alignment of 129S1/SvImJ and wt B6 genomes. Each color-coded bar represents an individual chromosome with its specific banding patterns shown. Each color-coded line represents a unique CTX event originating from that particular chromosome

Although we expected to observe an increased level of genomic complexity in NHEJ deficient B cell lymphomas, it is surprising to uncover such an astonishingly high level of genomic rearrangements (hundreds of events) ever detected in an experimental model. Previous studies showed that wt B cells had a very low level of translocations [12]. Therefore, to validate the presence of these structural rearrangements in G1XP lymphomas, we performed the whole genome NGS study using wt primary B cell samples derived from the same genetic background as the G1XP lymphomas. In this regard, we employed two types of activated B cells: 1) wt control sample one was collected from primary B cells activated with anti-CD40 and IL-4 for 4 days in the in vitro culture, which stimulated CSR and induced the generation of DSBs [27]; 2) wt control sample two was collected from primary GC B cells induced by *in vivo* immunization using specific antigens. Regardless of the types of activated B cells, we detected a remarkably high level of genomic rearrangements (210 and 243 CTXs respectively) in these wt control samples when we aligned our NGS data to mouse reference genome (Fig. 2a, Additional file 1: Table S1, mouse control 1 and mouse control 2). These data suggest that the widespread CTXs in G1XP lymphomas might be false positive and caused by difference in genetic background between our samples and mouse reference genome.

Since activated B cells may potentially harbor CTXs caused by DNA recombination events during antibody gene diversification [4, 29], we next employed the kidney genomic DNA sample from wt control to perform whole genome NGS. Indeed, we found that the wt control kidney DNA also exhibited 242 CTXs (Fig. 2b, Additional file 1: Table S1, kidney), thereby further corroborating that widespread genomic rearrangements in G1XP lymphomas are false positive that are not caused by deficiency of XRCC4 and/or p53, instead, by different genetic backgrounds of mouse strains. Of note, all CTXs identified and depicted in the Circo plots are distinct, non-overlapping events that are unique across all tissues including tumors and normal controls. All of the common CTXs that appeared twice between any given samples were removed, thus, our data suggest that such false positive CTXs seem to be random in nature.

### Validation of our NGS and analysis pipeline

To exclude the possibility that the complex genomic rearrangements (e.g. CTXs) we observed are artifacts introduced by our NGS analysis pipeline, we performed whole genome NGS using wt activated primary B cells isolated from pure C57BL/6J (B6) mice, which have the same genetic background as mouse reference genome (mm9). Then, we aligned our own whole genome NGS data from wt B6 activated B cells with mouse reference genome. In line with previous report [12], we detected a very low level of CTXs (only 9 events) in wt B6 B cells (Fig. 2c, Additional file 1: Table S1, WtB6).

**Fig. 3:**
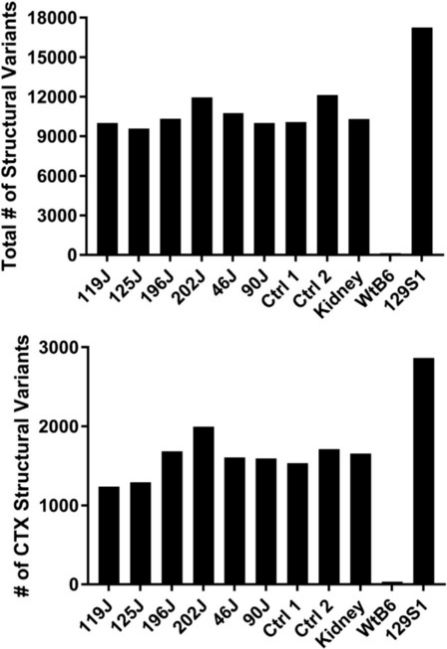
Candidate SV calls detected in samples of different genetic backgrounds before any filtering process. *Top*: the number of total SVs including DELs (deletions), CTXs (inter-chromosomal translocations) and others (see details in Additional file 2: Figure S1). *Bottom*: the number of CTXs in 10 sequenced samples including 6 tumor samples (119J, 125J, 196J, 202J, 46J, and 90J) and 4 control samples (control 1, control 2, kidney and wt B6) plus 129S1 whose sequences were downloaded from Sanger’s Institute (see details in Methods)

Before we applied the stringent filters, NGS samples derived from different genetic background (non-B6) harbored thousands of candidate SV calls including DELs and CTXs, when aligned with mouse reference genome (B6) (Fig. 3, Additional file 2: Figure S1). In sharp contrast, wt B6 sample which was sequenced by us harbored only 124 candidate SV calls (Additional file 2: Figure S1), when aligned with mouse reference genome (B6). After applying the filters and excluding any over-lapping CTXs that were identified in any other sample among the ten samples sequenced, we only detected 9 CTX events in wt B6 sample (Fig. 2c), which demonstrates a very low background and a relatively high accuracy of CREST algorithm, thereby validating our sequencing and analysis pipeline. In contrast, we still detected hundreds of CTXs in samples that were derived from different genetic backgrounds (non-B6) (Figs. 1 and 2a, b). Given that all NGS samples were processed in the exactly same manner, we attributed the vast difference in CTX numbers to the different genetic backgrounds of the samples, namely non-B6 vs B6.

Lastly, we aligned the NGS data of 129S1/SvImJ genome, which was downloaded from Sanger’s Institute [25], to mouse reference genome (mm9), then, employed CREST for SV detection. By simply aligning sequences from two mouse genomes (129S1/SvImJ vs B6), we uncovered an extremely high level of false positive genomic rearrangements (816 CTXs) (Figs. 2d and 3, Additional file 2: Figure S1 and Additional file 1: Table S1, 129S1). The reason for us to choose 129S1/SvImJ genome is because: (1) 129-related strains represent the genetic backgrounds on which numerous knockout mice have been generated [30]; (2) *Xrcc4* and *p53* conditional knockout mice were derived from 129-related strains (see Methods). Therefore, our data show that variation in genetic background may contribute to a high level of false positive genomic rearrangements, in particular, CTXs.

**Fig. 4:**
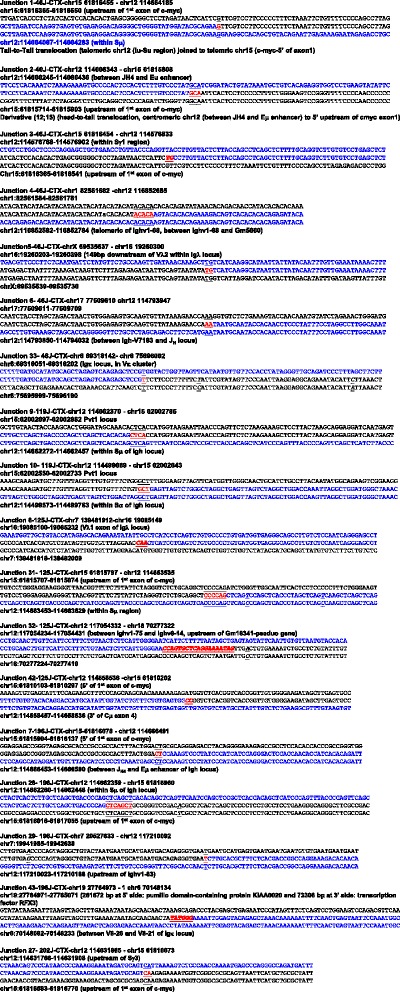
Sequence analyses of translocation breakpoints involving *Ig* loci. NGS data are aligned with mouse genomic sequences (mm9) via NCBI blast and Lasergene software. The sequences of *Ig* loci are in *blue* while the sequences of translocation partners are in *black*. Micro-homology (MH) is identified as the longest region with perfect homology between the *top* and *bottom* sequences. MH: *red text underlined* at the breakpoints with the homologous sequences on *top* and *bottom* underlined. Insertions: *red bold italic* text at the breakpoints. Point mutations: italic and underlined text

**Fig. 5:**
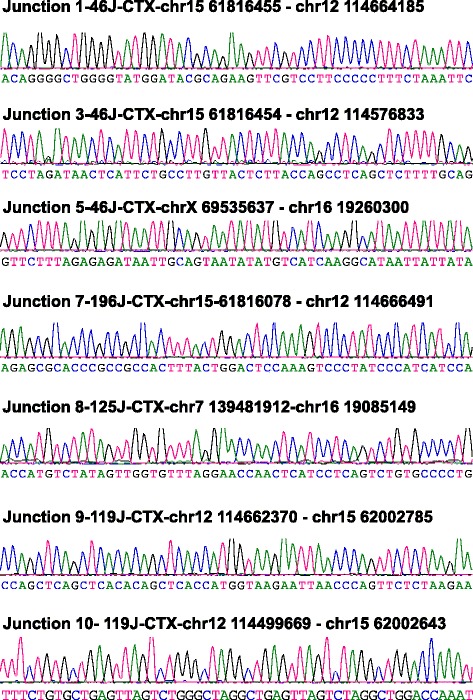
Sequencing results of translocations validated by PCR assays. Tumor DNA samples were employed for PCR assay using primers in the translocated loci. PCR products were purified, subcloned and sequenced. A fraction of translocations were validated by this methodology

Notably, some of the CTX events identified in G1XP lymphomas are indeed authentic translocations caused by NHEJ deficiency that were validated with independent methodology including FISH or PCR assays, such as the reciprocal *Igh-c-myc* translocations [28] (Figs. 4 and 5). Majority of these translocations harbor micro-homology (MH) (Fig. 4), which is an indicator of alternative end-joining [6]. However, the issue of different genetic background complicated the identification of such authentic CTXs in these B cell lymphoma samples. While this issue might be resolved by generating cohorts with the same genetic background as mouse reference genome, it appears to be more difficult to resolve such problems with human cancer genome sequencing.

## Discussion

Whole genome NGS approach potentially provides an exciting opportunity to discover chromosomal rearrangements in cancer genomes given that there were no previous systematic approaches to study cancers with complex genomes. In this regard, whole genome NGS has been increasingly employed to reveal the genomic landscape of tumor samples [31], including lymphoid malignancies [32] and solid tumors [19, 33–36]. For instance, recent whole genome sequencing studies show that the genome of human B cell lymphomas exhibits a high level of genomic complexity, including translocations, deletions, as well as indications of chromothripsis [37]. Several prior studies also identified complex structural rearrangements in different types of solid tumors [19, 33–36]. Thus, we attempted to employ this novel approach to analyze the genomic landscape of our newly developed B cell lymphomas [28].

Surprisingly, we found that widespread false positive genomic rearrangements (CTXs) can be generated by simply aligning sequences from different genetic backgrounds of mouse. This phenomenon is likely attributed to the possibility that reference genomes are different for various mouse strains (e.g. 129S1/SvImJ vs C57BL/6). Consistently, previous studies report that structural variations exist on a large scale in different mouse strains [25]. This study nicely revealed a global picture of mouse genomes from 17 different inbred strains [25]. Our current study highlighted the issue in the context of a specific application of whole-genome NGS, namely, identifying chromosomal translocations. High-throughput translocation sequencing has been employed to identify chromosomal translocations using mouse primary B cells [38, 39]. Among prior studies, NGS data inevitably has to be mapped and aligned with mouse reference genome (mm9) which is derived from B6 genetic background. However, mouse genetic background has not been considered to be an influential factor when interpreting NGS data. On the basis of our current study, we suggested that genomic DNA samples from pure B6 background are preferred when utilizing whole-genome NGS for chromosomal translocation studies in mice.

In the scenario of human patients, given that human populations are generally out-bred, it is impossible to exclude the influence of genetic background. In fact, the heterogeneity of human populations in genome variation has already been recognized [18]. However, these prior studies tend to focus on structural variations that result in a change in DNA dosage (copy number variants), in particular, deletions [40, 41]. For example, an analytical framework has been presented to characterize genome deletion polymorphism in populations using NGS data distributed across hundreds or thousands of human genomes. While this population genetic approach may be useful for identifying deletion variants involved in complex diseases [40, 41], it does not seem to be applicable to cancer genome sequencing.

Many of the prior cancer genome sequencing studies inevitably involved the mapping and alignment step for data analysis, which means that NGS data was mapped and aligned with human reference genome (NCBI Build 36 or other version) [19]. However, our results showed that simply aligning sequences from different genetic backgrounds of mouse generated a high level of false positive CTXs. Such a high level of background CTXs is only preventable when NGS data is obtained from mouse whose genetic background is the same as current available mouse reference genome (in B6 background). Thus, mapping and alignment with reference genome might not be a preferred method for analyzing whole-genome NGS data obtained from a genetic background that is different from reference genome.

Therefore, due to the uniqueness of every cancer genome, the heterogeneity of individual cancer cells and the difficulty of correctly mapping rearranged sequences and distinguishing them between cancers and normal control tissues, we suggest that *de novo* assembly of cancer genomes and matched controls is likely to become the preferred approach to analyze NGS data. However, this approach is much more computationally complex and technically challenging [19], which also requires a higher in-depth coverage of NGS data and a more cost-effective platform to obtain a large amount of NGS data from an individual cancer and its matched control. In this regard, single cell whole-genome NGS might be able to resolve this issue given that NGS data can be individually collected from hundreds or thousands of single cancer cell [31].

## Conclusion

Our studies showed that widespread false positive CTXs can be generated by simply aligning sequences from different genetic backgrounds of mouse. Thus, we conclude that it is necessary to consider the influence of genetic background on the level of genomic instability when performing whole genome NGS to discover chromosomal translocations.

## Additional files

Additional file 1: Table S1 The numbers of CTXs in each sample after filtering process and chromosome coordinates of all CTXs. 10 sequenced samples include 6 tumor samples (119J, 125J, 196J, 202J, 46J, and 90J) and 4 control samples (mouse control 1, mouse control 2, kidney and wt B6) plus 129S1 whose sequences were downloaded from Sanger’s Institute (see details in Methods). (XLSX 541 kb)
Additional file 2: Figure S1. Numbers of Candidate SV Calls Before Filtering Process. The numbers of candidate SV calls detected in 10 samples of different genetic backgrounds before any filtering process. The numbers of total SVs include ITX (intra-chromosomal translocations), DELs (deletions), INV (inversions), INS (insertions), and CTXs (inter-chromosomal translocations) in 10 sequenced samples, including 6 tumor samples (119J, 125J, 196J, 202J, 46J, and 90J) and 4 control samples (control 1, control 2, kidney and wt B6) plus 129S1 whose sequences were downloaded from Sanger’s Institute (see details in Methods). (PDF 361 kb)

## Abbreviations

NHEJ: Non-homologous end-joining (NHEJ)
CSR: Class switch recombination
NGS: Next generation sequencing
SV: Structural variation
CTX: Inter-chromosomal translocations
GC: Germinal center

## References

[1] Alt FW, Zhang Y, Meng FL, Guo C, Schwer B (2013). Mechanisms of programmed DNA lesions and genomic instability in the immune system. Cell.

[2] Nussenzweig A, Nussenzweig MC (2010). Origin of chromosomal translocations in lymphoid cancer. Cell.

[3] Honjo T, Kinoshita K, Muramatsu M (2002). Molecular mechanism of class switch recombination: linkage with somatic hypermutation. Annu Rev Immunol.

[4] Chen Z, Wang JH (2014). Generation and repair of AID-initiated DNA lesions in B lymphocytes. Front Med.

[5] Lieber MR (2010). The mechanism of double-strand DNA break repair by the nonhomologous DNA end-joining pathway. Annu Rev Biochem.

[6] Boboila C, Alt FW, Schwer B (2012). Classical and alternative end-joining pathways for repair of lymphocyte-specific and general DNA double-strand breaks. Adv Immunol.

[7] Boboila C, Jankovic M, Yan CT, Wang JH, Wesemann DR, Zhang T, Fazeli A, Feldman L, Nussenzweig A, Nussenzweig M (2010). Alternative end-joining catalyzes robust IgH locus deletions and translocations in the combined absence of ligase 4 and Ku70. Proc Natl Acad Sci U S A.

[8] Boboila C, Yan C, Wesemann DR, Jankovic M, Wang JH, Manis J, Nussenzweig A, Nussenzweig M, Alt FW (2010). Alternative end-joining catalyzes class switch recombination in the absence of both Ku70 and DNA ligase 4. J Exp Med.

[9] Difilippantonio MJ, Petersen S, Chen HT, Johnson R, Jasin M, Kanaar R, Ried T, Nussenzweig A (2002). Evidence for replicative repair of DNA double-strand breaks leading to oncogenic translocation and gene amplification. J Exp Med.

[10] Difilippantonio MJ, Zhu J, Chen HT, Meffre E, Nussenzweig MC, Max EE, Ried T, Nussenzweig A (2000). DNA repair protein Ku80 suppresses chromosomal aberrations and malignant transformation. Nature.

[11] Wang JH, Alt FW, Gostissa M, Datta A, Murphy M, Alimzhanov MB, Coakley KM, Rajewsky K, Manis JP, Yan CT (2008). Oncogenic transformation in the absence of Xrcc4 targets peripheral B cells that have undergone editing and switching. J Exp Med.

[12] Wang JH, Gostissa M, Yan CT, Goff P, Hickernell T, Hansen E, Difilippantonio S, Wesemann DR, Zarrin AA, Rajewsky K (2009). Mechanisms promoting translocations in editing and switching peripheral B cells. Nature.

[13] Yan CT, Boboila C, Souza EK, Franco S, Hickernell TR, Murphy M, Gumaste S, Geyer M, Zarrin AA, Manis JP (2007). IgH class switching and translocations use a robust non-classical end-joining pathway. Nature.

[14] Ochi T, Wu Q, Blundell TL (2014). The spatial organization of non-homologous end joining: from bridging to end joining. DNA Repair (Amst).

[15] Wang JH (2012). Mechanisms and impacts of chromosomal translocations in cancers. Front Med.

[16] Dalla-Favera R, Bregni M, Erikson J, Patterson D, Gallo RC, Croce CM (1982). Human c-myc onc gene is located on the region of chromosome 8 that is translocated in Burkitt lymphoma cells. Proc Natl Acad Sci U S A.

[17] Rowley JD (2001). Chromosome translocations: dangerous liaisons revisited. Nat Rev Cancer.

[18] Conrad DF, Hurles ME (2007). The population genetics of structural variation. Nat Genet.

[19] Meyerson M, Gabriel S, Getz G (2010). Advances in understanding cancer genomes through second-generation sequencing. Nat Rev Genet.

[20] Casola S, Cattoretti G, Uyttersprot N, Koralov SB, Seagal J, Hao Z, Waisman A, Egert A, Ghitza D, Rajewsky K (2006). Tracking germinal center B cells expressing germ-line immunoglobulin gamma1 transcripts by conditional gene targeting. Proc Natl Acad Sci U S A.

[21] Jonkers J, Meuwissen R, van der Gulden H, Peterse H, van der Valk M, Berns A (2001). Synergistic tumor suppressor activity of BRCA2 and p53 in a conditional mouse model for breast cancer. Nat Genet.

[22] Wang J, Mullighan CG, Easton J, Roberts S, Heatley SL, Ma J, Rusch MC, Chen K, Harris CC, Ding L (2011). CREST maps somatic structural variation in cancer genomes with base-pair resolution. Nat Methods.

[23] Wang Y, Waters J, Leung ML, Unruh A, Roh W, Shi X, Chen K, Scheet P, Vattathil S, Liang H (2014). Clonal evolution in breast cancer revealed by single nucleus genome sequencing. Nature.

[24] Xie T, Cho YB, Wang K, Huang D, Hong HK, Choi YL, Ko YH, Nam DH, Jin J, Yang H (2014). Patterns of somatic alterations between matched primary and metastatic colorectal tumors characterized by whole-genome sequencing. Genomics.

[25] Keane TM, Goodstadt L, Danecek P, White MA, Wong K, Yalcin B, Heger A, Agam A, Slater G, Goodson M (2011). Mouse genomic variation and its effect on phenotypes and gene regulation. Nature.

[26] Krzywinski M, Schein J, Birol I, Connors J, Gascoyne R, Horsman D, Jones SJ, Marra MA (2009). Circos: an information aesthetic for comparative genomics. Genome Res.

[27] Chen Z, Ranganath S, Viboolsittiseri SS, Eder MD, Chen X, Elos MT, Yuan S, Hansen E, Wang JH (2014). AID-initiated DNA lesions are differentially processed in distinct B cell populations. J Immunol.

[28] Chen Z, Elos MT, Viboolsittiseri SS, Gowan K, Leach SM, Rice M, Eder MD, Jones K, Wang JH (2016). Combined deletion of Xrcc4 and Trp53 in mouse germinal center B cells leads to novel B cell lymphomas with clonal heterogeneity. J Hematol Oncol.

[29] Wang JH (2013). The role of activation-induced deaminase in antibody diversification and genomic instability. Immunol Res.

[30] Guan C, Ye C, Yang X, Gao J (2010). A review of current large-scale mouse knockout efforts. Genesis.

[31] Pikor L, Thu K, Vucic E, Lam W (2013). The detection and implication of genome instability in cancer. Cancer Metastasis Rev.

[32] Mullighan CG (2013). Genome sequencing of lymphoid malignancies. Blood.

[33] Stephens PJ, McBride DJ, Lin ML, Varela I, Pleasance ED, Simpson JT, Stebbings LA, Leroy C, Edkins S, Mudie LJ (2009). Complex landscapes of somatic rearrangement in human breast cancer genomes. Nature.

[34] Pleasance ED, Stephens PJ, O’Meara S, McBride DJ, Meynert A, Jones D, Lin ML, Beare D, Lau KW, Greenman C (2010). A small-cell lung cancer genome with complex signatures of tobacco exposure. Nature.

[35] Pleasance ED, Cheetham RK, Stephens PJ, McBride DJ, Humphray SJ, Greenman CD, Varela I, Lin ML, Ordonez GR, Bignell GR (2010). A comprehensive catalogue of somatic mutations from a human cancer genome. Nature.

[36] Campbell PJ, Stephens PJ, Pleasance ED, O’Meara S, Li H, Santarius T, Stebbings LA, Leroy C, Edkins S, Hardy C (2008). Identification of somatically acquired rearrangements in cancer using genome-wide massively parallel paired-end sequencing. Nat Genet.

[37] Morin RD, Mungall K, Pleasance E, Mungall AJ, Goya R, Huff RD, Scott DW, Ding J, Roth A, Chiu R (2013). Mutational and structural analysis of diffuse large B-cell lymphoma using whole-genome sequencing. Blood.

[38] Klein IA, Resch W, Jankovic M, Oliveira T, Yamane A, Nakahashi H, Di Virgilio M, Bothmer A, Nussenzweig A, Robbiani DF (2011). Translocation-capture sequencing reveals the extent and nature of chromosomal rearrangements in B lymphocytes. Cell.

[39] Chiarle R, Zhang Y, Frock RL, Lewis SM, Molinie B, Ho YJ, Myers DR, Choi VW, Compagno M, Malkin DJ (2011). Genome-wide Translocation Sequencing Reveals Mechanisms of Chromosome Breaks and Rearrangements in B Cells. Cell.

[40] Handsaker RE, Korn JM, Nemesh J, McCarroll SA (2011). Discovery and genotyping of genome structural polymorphism by sequencing on a population scale. Nat Genet.

[41] Mills RE, Walter K, Stewart C, Handsaker RE, Chen K, Alkan C, Abyzov A, Yoon SC, Ye K, Cheetham RK (2011). Mapping copy number variation by population-scale genome sequencing. Nature.

