# Supplementary figures and images for: Unexpected effects of different genetic backgrounds on identification of genomic rearrangements via whole-genome next generation sequencing
Source: BMC Genomics. 2016 Oct 21;17:823. doi: 10.1186/s12864-016-3153-9 (PMC5075209; doi:10.1186/s12864-016-3153-9)

## Numbers of Candidate SV Calls Before Filtering Process

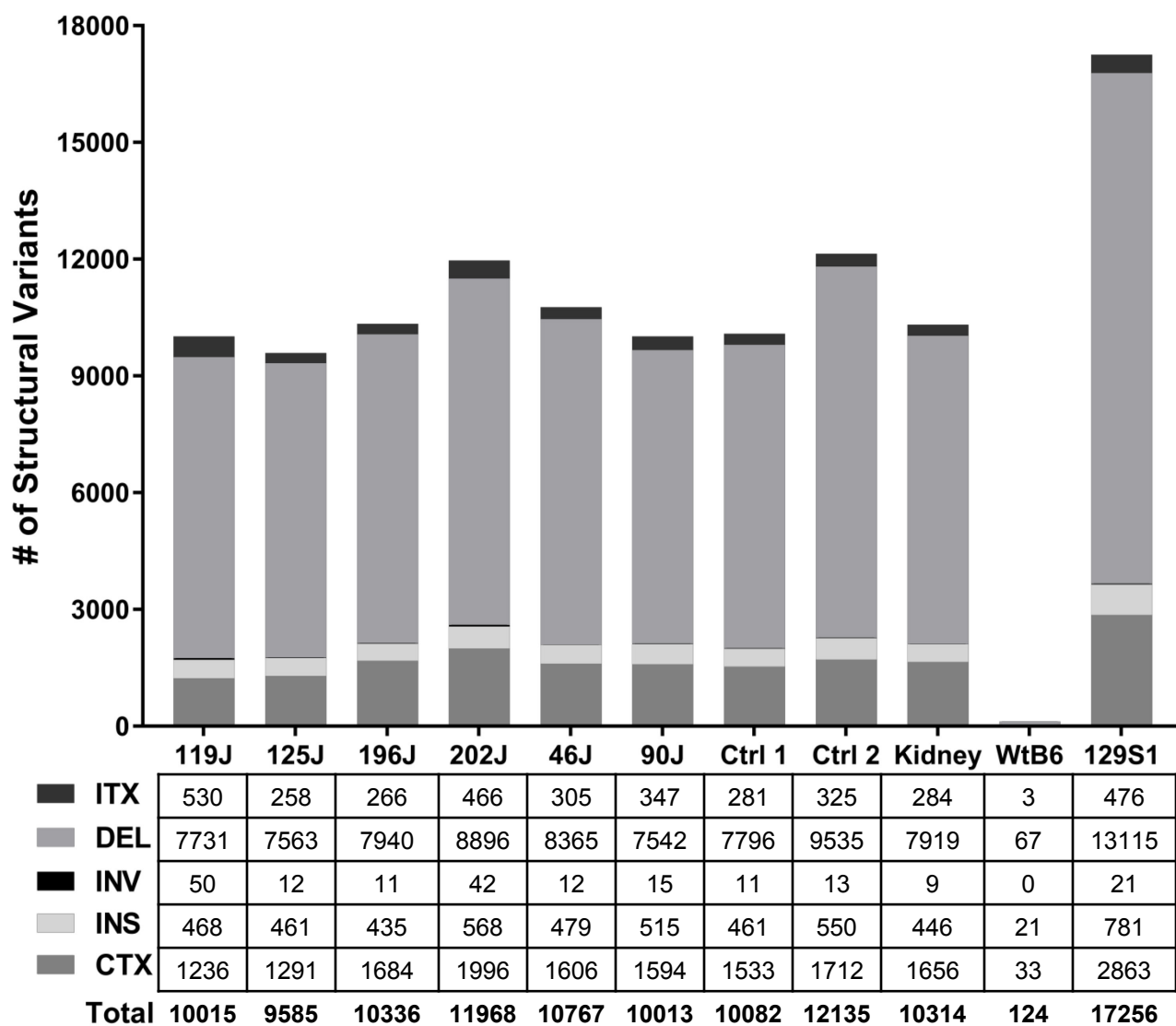

Supplement: Additional file 2: Figure S1. — Numbers of Candidate SV Calls Before Filtering Process. The numbers of candidate SV calls detected in 10 samples of different genetic backgrounds before any filtering process. The numbers of total SVs include ITX (intra-chromosomal translocations), DELs (deletions), INV (inversions), INS (insertions), and CTXs (inter-chromosomal translocations) in 10 sequenced samples, including 6 tumor samples (119J, 125J, 196J, 202J, 46J, and 90J) and 4 control samples (control 1, control 2, kidney and wt B6) plus 129S1 whose sequences were downloaded from Sanger’s Institute (see details in Methods). (PDF 361 kb) [file 12864_2016_3153_MOESM2_ESM.pdf]
